# Body Composition and Metabolic Status of Italian and Spanish University Students: Relationship with Fruit and Vegetable Consumption

**DOI:** 10.3390/nu14163296

**Published:** 2022-08-11

**Authors:** Paola Aiello, Ilaria Peluso, Silvia Di Giacomo, Antonella Di Sotto, Débora Villaño Valencia

**Affiliations:** 1 Department of Physiology and Pharmacology “V. Erspamer”, La Sapienza University of Rome, 00185 Rome, Italy; 2 Health Sciences PhD Program, Universidad Católica de Murcia UCAM, Campus de los Jerónimos n°135, 30107 Guadalupe, Spain; 3 Council for Agricultural Research and Economics, Research Center for Food and Nutrition, Via Ardeatina 546, 00178 Rome, Italy; 4 “Nutrición, Estrés Oxidativo y Biodisponibilidad” Research Group, Faculty of Health Sciences, School of Pharmacy, Universidad Católica de Murcia UCAM, Campus de los Jerónimos n°135, 30107 Guadalupe, Spain; 5 “Producción Animal, Nutrición y Ciencia de los Alimentos” Research Group, Department of Agronomy, Biotechnology and Food Science, Universidad Pública de Navarra (UPNA), Campus de Arrosadía, 31006 Pamplona, Spain

**Keywords:** Mediterranean diet, body composition, physical activity, metabolic status, bioelectrical impedance analysis, polyphenols

## Abstract

Most university students do not follow recommendations for fruit and vegetable intake, with a consequent increase in the prevalence of cardiovascular disease (CVD) risk factors. The aim of this study was to compare obesity prevalence and biomarkers of metabolic status between Italian and Spanish university students, in relation with the consumption of fruits and vegetables. Food consumption, adherence to a Mediterranean diet (MD), level of physical activity (PA), blood glucose, total cholesterol, triglycerides and ketones, blood pressure, and body composition were evaluated. Among CVD risk factors, only glucose was significantly higher in Spaniards (SP), and only 3.1% of SP presented ketosis. SP had a higher percentage of energy from fat. Although adherence to MD and fruit and vegetable consumption did not differ between Italians and SP, students who consumed at least four servings of fruit and vegetables (FV group) showed better values for pressure and metabolic parameters than the no FV group. We observed an association between consumption of fruit and PA. Students who consumed more vegetables than fruit reported a better body composition profile and lower glucose concentrations. As previously suggested, in addition to PA, two servings of fruit and three servings of vegetables per day should be recommended.

## 1. Introduction

Over the years, several studies have highlighted the beneficial effect exerted by the Mediterranean diet (MD) on different diseases, among which is metabolic syndrome (MetS). Additionally, adherence to this dietary regime has been associated with a lower incidence of metabolic risk factors such as cardiovascular diseases (CVDs), type 2 diabetes mellitus (T2DM), and cancer [1]. Notably, these beneficial effects are ascribable to the antioxidant and anti-inflammatory properties of MD foods, such as olive oil, nuts, vegetables, and fruits, and seem to be connected to their polyphenol content [2].

Over the last 20 years, there has been an increase in obesity rates among college students [3,4,5]. Moreover, most university students do not follow national recommendations [6]. It has been shown that less than 50% of students eat at least one serving of fruit per day, and less than 1 in 4 eat at least two servings of vegetables per day, with a consequent deviation towards a Western diet [7] and a high prevalence of overweight (OW) and obesity in countries that should adopt a traditional MD [8,9]. Among university students from 24 countries, 80.5% had an inadequate fruit and vegetable intake and 23.1% were OW/obese (OB) [10].

Compliance with the recommendations of the MD pyramid has been evaluated among Italians (IT), and the regular consumption of fruits and vegetables was significantly more common in females, and it increased with age and education (84.7% of those with a university degree) [11]. A study carried out at Veracruz University reported that young people aged 17–24 adopted an improper lifestyle, with 37% of students being OW or OB. They stopped exercising and skipped breakfast, with a consequent increase in insulin resistance, associated with the development of T2DM [12]. Ketosis-prone T2DM is a subtype of T2DM with traits of OW or obesity, with hyperglycemia and elevated serum triglycerides (TG) levels [13,14,15]. In addition, individuals with obesity, particularly abdominal obesity, commonly exhibit high TG or total cholesterol (TC) values [13,14,15].

In a recent systematic review, it has been reported that alcohol consumption and low consumption of fruits and vegetables were among the main behaviors associated with weight gain [16]. We previously observed in Italian and Spanish university students a low risk of excessive alcohol consumption, whereas 37.6% of the sample had poor adherence to MD from the alternate Mediterranean diet (aMED) score (including vegetables, fresh fruits, dried fruits, wholegrain cereals, pulses, fish, and olive oil) [17].

In this context, the present study was aimed at comparing the obesity prevalence and biomarkers of metabolic status between Italian and Spanish university students, in relation to the consumption of fruits and vegetables [18], in order to highlight possible differences between two Mediterranean basin populations and increase our knowledge about the preventing properties of the MD diet towards MetS. Furthermore, we hypothesized that Italian and Spanish students might have different eating habits and lifestyles, assuming that subjects with a high adherence to the MD have a better metabolic status.

## 2. Materials and Methods

### 2.1. Study Design, Recruitment, and Data Collection

This study was carried out on a sub-group of participants from a previous study about the lifestyle and dietary habits of university students [17]. Non-working undergraduate and doctoral students aged between 18 and 35 years were recruited at La Sapienza University of Rome and at the Catholic University of Murcia. All the volunteers included in the study signed the informed consent, accompanied by an informative note, and the recruiter assigned them an alphanumeric code to guarantee privacy during the data processing and analysis phases (all details about recruitment and protocol are available online at https://www.clinicaltrials.gov/ct2/show/NCT04099420 (accessed on 23 May 2022). Online questionnaires were administered to collect information about dietary habits, level of physical activity (PA), and lifestyle of the subjects. Moreover, students were evaluated with clinical parameters of metabolic status (glycaemia, TC, TG, and ketones), and underwent anthropometric, impedance, and blood pressure measurements. The presence of risk factors for MetS was assessed: waist circumference (WC, ≥88 cm in women, ≥102 cm in men), elevated TG (≥150 mg/dL) or drug treatment for hypertriglyceridemia, high blood pressure (BP, systolic ≥ 130 mmHg or diastolic ≥ 85 mmHg) or hypertensive drug treatment, and high fasting glucose (>100 mg/dL) or drug treatment for hyperglycemia [19,20,21]. Participants filled in a 7-day food diary to assess energy and macronutrient intake.

### 2.2. Adherence to Mediterranean Diet

As described in the previous study [17], to evaluate the grade of adherence to MD, three different questionnaires were administered to the volunteers: Mediterranean diet score (MDS-14) [22], Mediterranean score (MED-55) [23,24], and a questionnaire to measure Mediterranean diet (QueMD) [25]. Furthermore, the aMED score, ranging from 0 (minimal adherence to MD) to 9 (maximal adherence), was calculated from QueMD assigning 1 point to volunteers consuming red and processed meat (≤1–3/week), dried fruits (≥2/week), wholegrain cereals (≥1/day), pulses and fish (≥2/week), olive oil (≥3/day), for men drinking 1–2 glasses of wine per day (corresponding to 125–250 mL) or women drinking a limited amount of wine (1/2–1 glass/day, corresponding to 62.5–125 mL) [26]. Moreover, 1 point was also assigned to those reporting consumptions above the average levels for fruits (≥2/day) and vegetables (≥2/day) [26]. From this scoring protocol, subscores for fruit (F-QueMD) and vegetable (V-QueMD) intake have been used in the present study in order to evaluate their relationship with obesity and MetS markers.

### 2.3. Physical Activity

The International Physical Activity Questionnaire (IPAQ) [27] was used to evaluate the PA level. The IPAQ (short form) [27] includes items assessing the frequency and duration of PA in three ranges of intensity: intense PA (8.0 metabolic equivalent of tasks: METs), moderate PA (4.0 METs), and walking at a fast (3.3 METs), moderate (3.0 METs), and slow (2.5 METs) pace [27]. Based on collected data about the frequency and duration of PA, energy expenditure (expressed as MET-min/week) has been estimated. One MET is the rate of energy expenditure at rest, and it is approximately equal to 3.5 mL O_2_ kg^−1^ min^−1^ in adults. According to the Italian Society of Endocrinology (SIE), IPAQ allows population to be classified into three PA levels: low (the lowest level of PA, less than 700 METs-min/week), moderate (total PA between 700 and 2519 METs-min/week) and high (total PA of at least 2520 METs-min/week).

### 2.4. Anthropometric, Body Composition and Blood Pressure Measurements

Height was measured with the SECA 217 portable stadiometer, with the measure approximated to the nearest 0.1 cm [28], whereas WC and hips circumferences (HC) were measured with the anthropometric tape measure Anthroflex [28]. The waist-to-hips ratio (WHR) has been evaluated to describe the adipose tissue distribution and its related risks. It should be less than 0.90 for men and 0.85 for women [29].

To evaluate body composition, in particular body fat and muscle mass (MM), bioelectrical impedance analysis (BIA) has been used. Body weight, BMI, MM, and fat mass (FM) percentages were measured by the impedance balance OMRON BF511 with the subject in fasting conditions [30]. BMI classification according to the values for obesity judgement proposed by WHO has been described in the previous study [17].

In addition to BIA, body skinfold thickness was measured to the nearest 0.1 cm by the Harpenden caliper at biceps, triceps, subscapular, and supra-iliac areas on the right side of the body with the subject standing in a relaxed condition [28]. Then, FM percentage was calculated from body density using Siri’s equation [31].

BP was measured with Omron X3 Comfort Upper Arm Blood Pressure Monitor.

### 2.5. Capillary Blood Sampling

Clinical parameters of metabolic status were assessed from capillary blood samples. In particular, TC, TG, and glucose (Glu) blood levels were evaluated with the MultiCare In System [32]. To perform a measurement with the MultiCare In System, it was necessary to withdraw a drop of capillary blood using a lancing device that had six different penetration depths, allowing us to adjust the penetration depth to the texture of the volunteer skin [33]. MultiCare In has two different technologies: amperometric, with Glu electrode strips [34], and reflectometric, with TC [35] and TG strips [36]. To determine Glu concentrations, a volume of at least 0.5 μL of only capillary blood was needed [34]. Regarding TC and TG analysis, participants had to fast for at least 9 h and 12 h, respectively, and a volume of at least 10 μL of blood was required [35,36]. Furthermore, the Glucomen^®^ areo β-Keton Sensor kit [37] was used to evaluate blood ketones (Ket) concentration, and a volume of at least 0.8 μL of blood was required to detect Ket value.

### 2.6. Food Consumption Analysis

Food consumption was evaluated using a 7-day food diary. Participants were asked to write day-by-day quality and quantity of food consumed and to report how they felt before and after eating. A short training session was provided giving instructions in order to fill in the diary correctly. Furthermore, a photographic food atlas [38] was provided to subjects, which contained images of common dishes, so as to visually compare their dish at the time of evaluating the portion consumed. In this way, if the subject was unable to provide the exact weight, it would have been possible to estimate the quantity of food by means of the standardized portions. Data collected using the food diary made it possible to calculate food grams per day, total energy (kcal/day), and macronutrients (energy contribution percentage of proteins, lipids, and carbohydrates) through Food Composition tables of CREA-AN [39].

### 2.7. Statistics

A descriptive statistical analysis (averages, standard deviations, percentages) was first performed. Normal distribution of variables has been checked using the Shapiro–Wilk test. Categorical variables have been expressed as percentages, while continuous variables as means with standard deviation (SD) or medians with interquartile interval. Spearman correlations were used to evaluate relationships between variables. Bivariate analysis was conducted using an unpaired *t*-test and Mann–Whitney test for continuous variables, and chi-square test and Fisher′s exact test for categorical variables. Furthermore, results were analyzed using the Kruskal–Wallis one-way analysis of variance on ranks. Statistical analysis was performed using the Graph Pad software (GraphPad Prism 8 XML ProjectT, La Jolla, CA, USA), and variables with a *p*-value < 0.05 were considered statistically significant. Principal Component Analysis (PCA) was performed using PAST (PAleontological STatistics).

## 3. Results

### 3.1. Differences between Italians and Spaniards

Participants were university and doctoral students from both scientific (98.5%) and humanistic (1.5%) areas, with 87.9% and 12.1% living in the city and in a rural area, respectively. The two groups were comparable for gender distribution and smoking habit. Regarding BMI, WC, WHR, and waist-to-height ratio (WHtR), no significant differences have emerged between IT and Spaniards (SP), as well as in the prevalence of OW/OB and in mean values of BP.

Characteristics of participants and differences between Italy and Spain are reported in Table 1.

**Figure 1:**
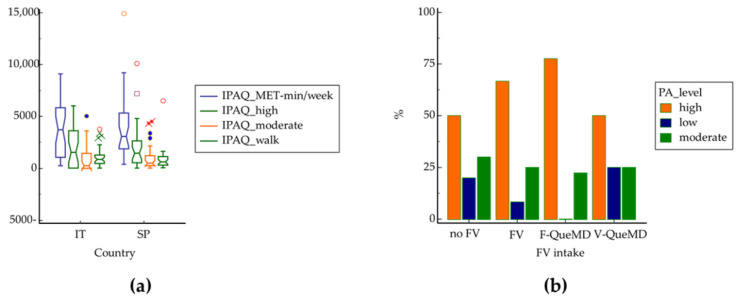
(**a**) Level of physical activity in Italians and Spaniards according to International Physical Activity Questionnaire (IPAQ); (**b**) prevalence of subjects who consume fruit and vegetables in relation to physical activity level. PA: physical activity; no FV: no consumption of fruit and vegetables (0 point for subscores F-QueMD and V-QueMD); FV: daily consumption of at least four servings of fruit and vegetables (1 point for F-QueMD and V-QueMD); F-QueMD: daily intake of at least two servings of fruit (1 point for F-QueMD and 0 point for V-QueMD); V-QueMD: daily consumption of at least two servings of vegetables (1 point for V-QueMD and 0 point for F-QueMD). Different colors of circles, squares and crosses represent the single values that deviate from the mean for each group of physical activity level.

Concerning lifestyle (PA and dietary habits), no significant differences were found in IPAQ (Figure 1a), but those who had high PA tended to consume more fruits and less vegetables than those who had low PA (Figure 1b).

As regards adherence to MD (Table 2), assessed with different questionnaires, no significant differences were found between IT and SP.

The average daily energy intake was significantly higher in SP than IT. As regards macronutrients, the daily energy intake of lipids, expressed as a percentage, was suitable for the Italian group, in line with the indications from the national guidance that recommends between 20% and 35% in adults, whereas the Spanish counterparts showed a significantly different consumption above recommendations [40]. Carbohydrate intake was adequate only in the Italian group. In fact, dietary guidelines recommend a carbohydrate intake corresponding to 50–60% of the total calories. No differences were observed in protein consumption between IT and SP. Dietary fiber intake was low, in fact a consumption of at least 25 g per day is suggested in the adult population, a quantity not reached by the two groups (Table 2).

The biochemical markers were evaluated by capillary sampling to determine Glu, TC, TG, and Ket plasma concentrations (Table 3).

Among the sample, 20.6% of IT and 50.0% of SP reported a prediabetes condition, manifested by glucose levels > 100 mg/dL and worthy of long-term monitoring, whereas 11.8% of IT and 18.8% of SP showed TC values ≥ 200 mg/dL, related to a moderate risk of experiencing CVD. As regards Ket concentrations, only 3.1% of Spanish students reported a ketosis condition.

### 3.2. Fruit and Vegetable Intakes and Metabolic Risk Factors Prevalence

**Figure 2:**
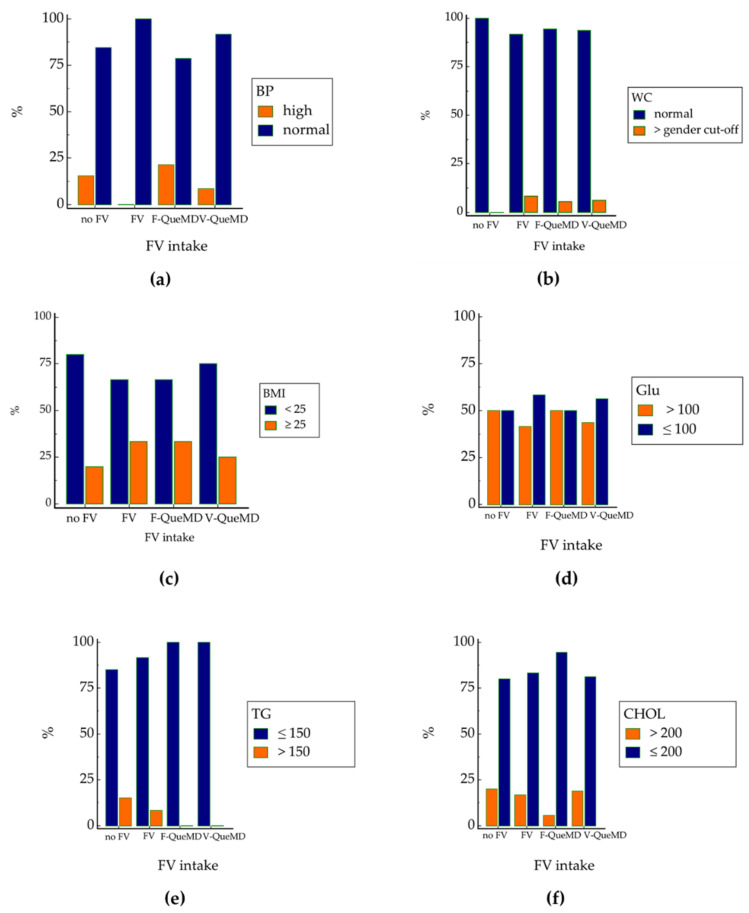
Prevalence of subjects with different intakes of fruit and vegetables in accordance with (**a**) blood pressure, (**b**) waist circumference, (**c**) body mass index, (**d**) plasma glucose, (**e**) triglycerides, and (**f**) cholesterol concentrations. no FV: no consumption of fruit and vegetables (0 point for subscores F-QueMD and V-QueMD); FV: daily consumption of at least four servings of fruit and vegetables (1 point for F-QueMD and V-QueMD); F-QueMD: daily intake of at least two servings of fruit (1 point for F-QueMD and 0 point for V-QueMD); V-QueMD: daily consumption of at least two servings of vegetables (1 point for V-QueMD and 0 point for F-QueMD); BP: blood pressure (high, systolic ≥130 mmHg); WC: waist circumference (gender cut off, ≥88 cm in women and ≥102 cm in men); BMI: body mass index; Glu: glucose; TG: triglycerides; CHOL: cholesterol.

In order to evaluate differences in body composition and metabolic status in relation to fruit and vegetable intake, the sample was divided into four groups (no FV n = 20, FV n = 18, F-QueMD n = 16, V-QueMD n = 12) on the basis of the subscores F-QueMD and V-QueMD (Figure 2). It emerged that those who consumed at least four servings of fruit and vegetables (FV group) showed better values for pressure and metabolic parameters than the no FV group, particularly for TG and TC levels. Furthermore, students who consumed more vegetables than fruit (higher V-QueMD score than F-QueMD) reported a better body composition profile and plasma Glu concentrations, whereas no differences have been observed between V-QueMD and F-QueMD groups for TG levels. Moreover, students who showed a higher intake of fruit (higher F-QueMD) have reported better levels of TC than the other groups.

Spearman correlations (Table 4) have been performed to evaluate relationships of F-QueMD and V-QueMD subscores with the other variables. It emerged that F-QueMD positively correlated with BMI, WHR, and PA (IPAQ), whereas V-QueMD was associated with the adherence to MD (MDS-14, QueMD and aMED).

**Figure 3:**
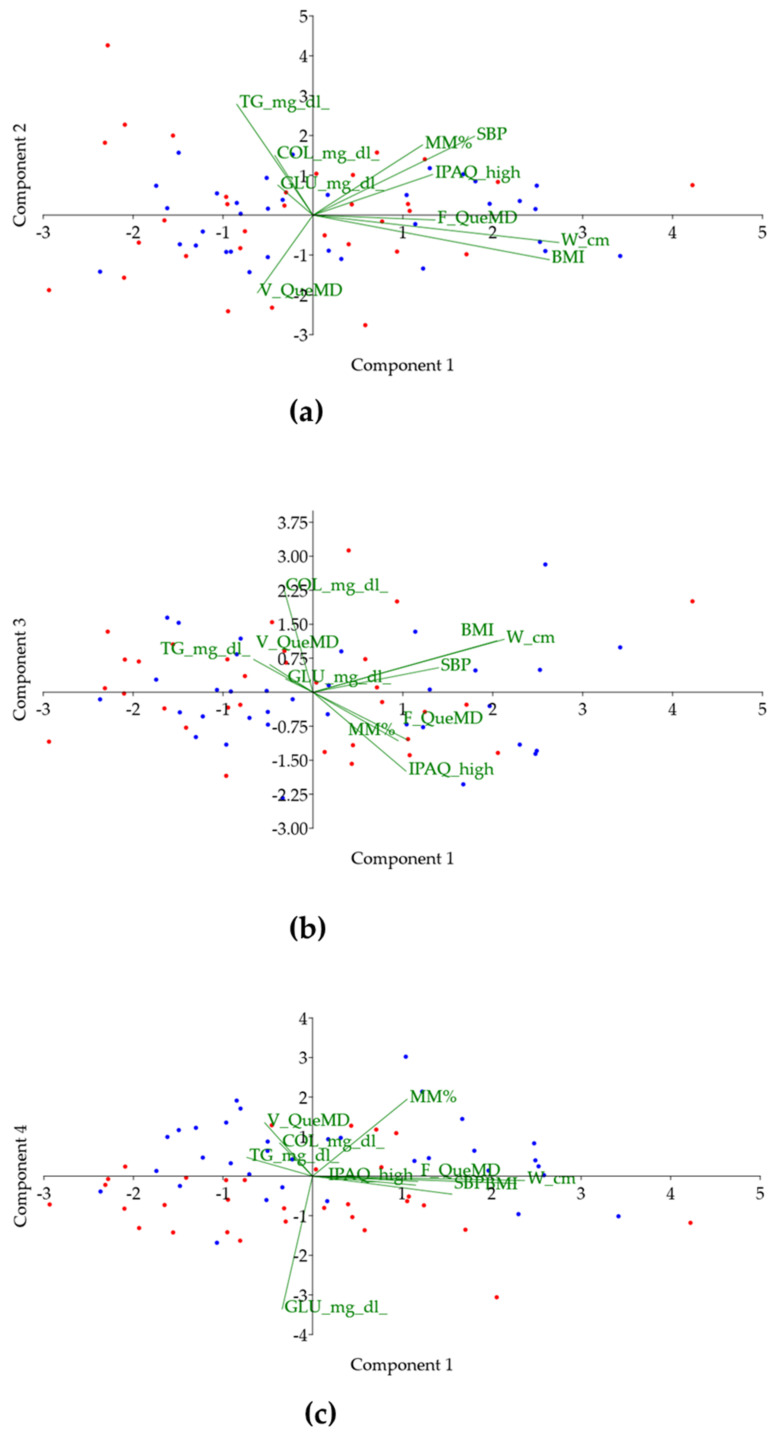
Principal component analysis (PCA). (**a**) Component 1 (PC1) vs. Component 2 (PC2); (**b**) Component 1 (PC1) vs. Component 3 (PC3); (**c**) Component 1 (PC1) vs. Component 4 (PC4). Italians and Spaniards are represented by blue and red points, respectively. TG: triglycerides; COL: cholesterol; GLU: glucose; %MM: muscle mass percentage; SBP: systolic blood pressure; IPAQ high: IPAQ from high intensity activities according to International Physical Activity Questionnaire; F-QueMD: subscore of QueMD representing score 0–1 (1 point for at least two servings of fruit); V-QueMD: subscore of QueMD representing score 0–1 (1 point for at least two servings of vegetables); W: waist circumference; BMI: body mass index.

In order to better evaluate the influence of fruit and vegetable intake on metabolic risk factors in IT and SP, a PCA was performed (Figure 3). Based on Eigenvalue > 1, PC1, PC2, PC3, and PC4 explained 24.2%, 14.6%, 12.6%, and 11.6% of variance, respectively. Among these, scatter plot PC1 versus PC4 (Figure 3c) seems the one that partly discriminates between IT (blue) and SP (red), although clear clusters were not evident. In particular, along the first component (Figure 3a) two macro groups emerged, one corresponding to clinical metabolic parameters and intake of vegetables (V-QueMD + TG + COL + GLU), and the other to high body composition and fruit consumption (F-QueMD + IPAQ + %MM + BMI + WC + SBP). As observed in Table 3, PCA confirmed that Glu was the major discriminant factor between IT and SP.

## 4. Discussion

This study aimed to compare body composition and metabolic status in Italian and Spanish university students in relation to fruit and vegetable consumption. In line with preceding studies [26,41,42,43,44,45,46,47], more than half of the sample reported an inadequate intake of fruit and vegetables, with a similar tendency among IT and SP. Furthermore, in accordance with the previous study [17], no significant differences emerged between IT and SP concerning the adherence to MD. It has been reported that the most common cardiovascular risk factors in undergraduate students with a low adherence to MD (11.3% reported a good compliance with MD) were smoking (20.6%), high TC (7.5%), and high BP (6.0%) [48]. In our sample, the prevalence of smoking was lower and the other CVD risk factors were related to fruit and vegetable consumption.

It has been found that the risk for excess body weight in the university students who reported not consuming fruits/vegetables daily was two to three times higher than in their peers who showed an adequate intake [49]. The authors of [49] reported a prevalence of OW of 39.1 %. In our study, about 22% (23.5% IT and 21.9% SP) of students were OW and the prevalence of OB was 5.9% and 3.1% in IT and SP, respectively. OB and OW prevalence were, respectively, similar and higher than those recently observed among university students in Kenya (13.9% OW and 4.3% OB) [47]. WHtR and WC are supposed to have greater discriminatory power compared to BMI [50], and are more sensitive than BMI as an early predictor of health-related risks [51]. In particular, WHtR is probably the most sensitive anthropometric index for the screening of MetS in Mediterranean populations, compared to both BMI and WC [52]. In young female science students from the Kingdom of Saudi Arabia, increased BMI values were associated with an elevated risk of developing MetS, as 41.4% of the OW and 44.8% of the OB students had three or more risk factors [53]. In contrast, among Medical University students in the south-east of Iran, BMI was a strong predictor of dyslipidemia, rather than the indexes of central obesity (WHR and WHtR) [54]. Furthermore, in Japanese university students, intra-abdominal fat accumulation was significantly associated with MetS markers, rather than WC alone [55]. Contrary to a recent study [47], a correlation was established between fruit consumption and BMI. Moreover, as reported by the authors of [47], students who consumed fruit and/or vegetables daily were more likely to be OW or OB. This could be explained by the consumption of a healthy diet, including an adequate intake of fruit and vegetables, in order to lose or prevent weight gain.

PA has been suggested as a means to reduce and control body fatness. In China, vegetable and fruit intake had a positive association with physical exercise and a negative association with fat mass index/free fat mass index (FMI/FFMI) ratio [56]. Overall, regular PA has proved to effectively reduce diverse health risk factors, especially those related to CVD and MetS [57,58]. MD pyramid gives importance not only to proper nutrition, but also generally to sport and PA [59]. No significant differences were observed in PA expressed as MET-min/week between IT and SP, but relationships between fruit and vegetable consumption and PA level were observed in the whole sample. In accordance with a previous larger study, involving 555 Spanish university students [60], we have found an association between the consumption of fruits and PA, but not with vegetable intake, contrary to other studies that have reported an association with both fruit and vegetable consumption [49,56].

From the analysis of the nutritional profile, a higher intake of lipids and a lower consumption of carbohydrates has been observed among SP than IT, with an adequate intake of protein for both groups.

According to the criteria of the National Cholesterol Education Program-Adult Treatment Panel III (NCEP-ATP III), in a previous study carried out in Yemeni students (11.7% OW), the overall prevalence of hypercholesterolemia and hypertriglyceridemia was 21.7% and 23.8%, respectively [61]. In China, the overall prevalence of dyslipidemia was 7.2% among university students [62]. In our sample, the prevalence of both high TG levels and ketosis were low, despite the high prevalence of OW/OB. In the students of the Faculty of Health of Universidad Santiago de Cali, the overall prevalence of high TG levels and hypercholesterolemia were 12.8% and 16.1%, respectively [63], and the consumption of alcoholic beverages was among the factors that were significantly associated with the risk of high TG levels, whereas for TC none was significant [63]. A prevalence of ketosis of 34.0% has been reported in Japanese alcoholic men (≥40 years), and that consumption of whiskey, hypoglycemia, and lower BMI were significant determinants of the development of ketosis [64]. In our study, the prevalence of ketosis was low, according to the low AUDIT scores previously reported in both country groups [17], but higher levels of Glu and Ket have been reported among SP, with the former being the major discriminant factor between the two populations. Prolonged fasting, alcoholism, and diets high in fat and low in carbohydrates can affect the values obtained [65]. Since a low risk of alcohol consumption has been reported in the previous larger study [17], such metabolic parameters values are more likely due to the different dietary habits between IT and SP and to the tendency to fast for extended periods. In Australia, although only 10% of students met fruit or vegetable recommendations, body composition and blood Glu concentrations were largely normal [66]. Moreover, in non-obese Japanese male university students fasting blood glucose was within normal glucose tolerance ranges and TG values did not correlate with either BMI or WC [67].

The study has some limitations, and our results should be interpreted carefully. The major limitation is the limited sample size. Moreover, since respondents were from central Italy and southern Spain because of the university locations, the results of this study may not be generalizable to all Italian and Spanish university students. Furthermore, the COVID-19 pandemic has represented a limitation to this study since it limited recruitment, and it has induced strong changes in the lifestyle and eating habits of university students, that could explain the differences that emerged with American students, in particular regarding alcohol consumption. However, a higher calorie consumption has emerged among SP that influenced metabolic parameters, in addition to a higher intake of fruit that mostly affects body composition.

## 5. Conclusions

The different relationship of fruit and vegetable intake with obesity was in line with the previous finding that the daily intake of three servings of vegetables and two servings of fruit was associated with lower mortality, and a higher intake was not associated with additional risk reduction [68]. Furthermore, the relationship between nutrition, health, and fitness constitutes a value of fundamental importance to achieve a better quality of life, health promotion, and disease prevention [69]. PA is a determining factor for energy expenditure which is essential for weight control [69]. Therefore, both nutrition and PA are important factors affecting body composition.

## Figures and Tables

**Table 1:** Students′ characteristics.

Variable	IT (*n* = 34)	SP (*n* = 32)	*p*
Age (years) *	25.9 ± 3.6	24.0 ± 3.7	0.0382
**Gender** ***Males (%)***	44.1	43.8	>0.999
**Smokers (%)**	17.7	12.5	0.1030
**Height (m)**	1.69 ± 0.09	1.71 ± 0.09	0.3940
**Weight (kg)**	62.2 (54.4–81.6)	67.0 (57.1–76.4)	0.9416
**WC (cm)**	72.4 (67.0–85.5)	74.5 (67.7–82.0)	0.9365
**HC (cm)**	95.0 (90.6–100.0)	96.1 (91.8–101.2)	0.5988
**WHR**	0.77 (0.73–0.84)	0.78 (0.74–0.83)	0.8656
**WHtR**	0.43 (0.41–0.49)	0.44 (0.41–0.46)	0.5844
**BMI (kg/m^2^)**	22.1 (20.9–26.3)	22.7 (21.0–25.3)	0.9011
***Overweight (%)***	23.5	21.9	
***Obese (%)***	5.9	3.1	
**% FM**			
**Plicometry**	25.1 ± 15.5	20.7 ± 6.7	0.1600
**BIA**	26.5 ± 7.5	24.2 ± 9.3	0.2674
**% MM**	30.2 (28.8–37.9)	29.5 (22.1–36.7)	0.0815
**BP (mmHg)**			
***Systolic***	120.0 (110.0–120.0)	115.5 (105.5–120.0)	0.1730
***Diastolic***	75.0 (66.5–80.0)	70.0 (58.8–79.3)	0.0960

Categorical variables are expressed as percentage, and continuous variables as mean with standard deviation (Shapiro–Wilk Test passed), or as median with interquartile range (Shapiro–Wilk Test failed). IT: Italians; SP: Spaniards; WC: waist circumference; HC: hips circumference; WHR: waist-to-hips ratio; WHtR: waist-to-height ratio; BMI: body mass index; FM: fat mass; BIA: Bioelectrical Impedance Analysis; MM: muscle mass; BP: blood pressure. * *p* < 0.05.

**Table 2:** Adherence to Mediterranean diet and average daily macronutrient intake.

Variable	IT	SP	*p*
**MED-55**	34.1 ± 3.8	33.4 ± 7.4	0.6183
**MDS-14**	6.0 (5.0–8.0)	7.0 (6.0–9.0)	0.1384
**QueMD**	13.2 ± 3.5	13.8 ± 3.2	0.4694
**aMED**	4.0 (3.0–5.0)	3.0 (2.3–4.0)	0.7759
**Protein** ***% energy***	12.5 (10.0–15.5)	13.5 (11.8–17.3)	0.3277
**Lipids** ***% energy*** **	33.0 (29.0–37.8)	50.5 (44.8–65.0)	0.0011
**Carbohydrates** ***% energy*** **	49.7 ± 9.9	33.5 ± 12.4	0.0031
**Fiber (g)**	16.0 ± 5.8	20.7 ± 5.6	0.1217
Energy (Kcal) *	2156 (1828–2614)	3118 (2695–3333)	0.0240

Categorical variables are expressed as percentage, and continuous variables as mean with standard deviation (Shapiro–Wilk Test passed), or as median with interquartile range (Shapiro–Wilk Test failed). IT: Italians; SP: Spaniards; MED-55: Mediterranean score questionnaire; MDS-14: Mediterranean diet score questionnaire; QueMD: Questionnaire to measure Mediterranean diet; aMED: QueMD subscore alternate Mediterranean diet. * *p* < 0.05; ** *p* < 0.01.

**Table 3:** Metabolic status parameters.

Variable	IT	SP	*p*
Glu (mg/dL) ****	91.0 (81.8–98.3)	104.0 (99.0–110.8)	<0.0001
***Hypoglycemia (%)***	-	-	
***>100 mg/dL (%)***	20.6	50	
***Diabetes (%)***	-	-	
**TC (mg/dL)**	155.5 (138.3–175.5)	158.5 (130.0–189.5)	0.9770
***>200 mg/dL (%)***	11.8	18.8	
**TG (mg/dL)**	78.0 (63.3–92.0)	73.0 (51.3–101.0)	0.4607
***>150 mg/dL (%)***	2.9	9.4	
**Ket (mmol/L)**	0.2 (0.1–0.2)	0.2 (0.2–0.2)	0.0740
***Ketosis (%)***	-	3.1	
***Ketoacidosis (%)***	-	-	

Categorical variables are expressed as percentages, and continuous variables as median with interquartile range (Shapiro–Wilk Test failed). IT: Italians; SP: Spaniards. Glu: glucose (hypoglycemia, <60 mg/dL; cutoff for metabolic syndrome, between 101 and 125 mg/dL; diabetes, ≥126 mg/dL); TC: total cholesterol (moderate risk, between 200 and 239 mg/dL; high risk, ≥240 mg/dL); TG: triglycerides (moderate risk, between 150 and 199 mg/dL; high risk, between 200 and 499 mg/dL); Ket: ketones (ketosis, between 0.6 and 1.5 mmol/L; ketoacidosis, >1.5 mmol/L). **** *p* < 0.0001.

**Table 4:** Spearman correlations.

	F-QueMD	V-QueMD
**BMI**	0.244 (0.048)	
**WHR**	0.251 (0.042)	
**IPAQ MET-min/week**	0.257 (0.037)	
**MDS-14**		0.359 (0.003)
**QueMD**		0.483 (<0.001)
**aMED**		0.639 (<0.001)

Correlation coefficients (*p* value). F-QueMD: daily intake of at least two servings of fruit (1 point for F-QueMD and 0 point for V-QueMD); V-QueMD: daily consumption of at least two servings of vegetables (1 point for V-QueMD and 0 point for F-QueMD); BMI: body mass index; WHR: waist-to-hips ratio; IPAQ MET-min/week: Metabolic equivalent of tasks-min/week according to International Physical Activity Questionnaire; MDS-14: Mediterranean diet score questionnaire; QueMD: Questionnaire to measure Mediterranean diet; aMED: QueMD subscore alternate Mediterranean diet.

## Data Availability

Data are not available due to ethical restrictions (see Informed Consent Statement).
